# Analysis of Water Activity and Gloss of Stored Goat Cheeses According to Consumer Preferences and Tastes

**DOI:** 10.3390/foods13233789

**Published:** 2024-11-25

**Authors:** Łukasz K. Kaczyński

**Affiliations:** Department of Dairy and Process Engineering, Faculty of Food Science and Nutrition, Poznań University of Life Sciences, ul. Wojska Polskiego 31, 60-624 Poznań, Poland; lukasz.kaczynski@up.poznan.pl

**Keywords:** gloss, water activity, goat’s cheese, color, consumers, ecologic

## Abstract

Packaging is an integral part of every food product, especially cheese. An important goal is to protect the product from spoiling and drying out. Two types of cheese were tested: soft goat’s cheese and hard goat’s cheese. They were evaluated for gloss, water activity, and colour. The aim of the research was to assess changes in the water activity of goat cheese in correlation with changes in gloss and color during storage in various forms of packaging, depending on consumer habits. The research problem was based on consumer observations regarding the repackaging of dairy products, including goat’s cheese. Consumers have reported such a problem in previous studies. The question was asked: will it be necessary in the future to indicate to the consumer the appropriate form of repackaging for a given goat’s cheese? It was shown that the best packaging for storing open feta-type goat salad cheeses was aluminum foil and hard goat cheeses in the producer’s packaging. The method of storage only affects the change in gloss in the case of goat salad cheese and parameter a* hard cheese. At the same time, the need was noted to develop appropriate packaging that would serve to protect the product from spoilage and would not pose a threat to the natural environment after being thrown into the trash. Wrapping soft goat cheeses in cellulose fiber paper reduced water activity by 5% after 14 days of storage but did not encourage re-consumption. The key task for future research is, therefore, to carry out regular consumer surveys. Therefore, it is necessary to choose (develop) a packaging that would preserve the original quality of the cheeses when stored in these conditions.

## 1. Introduction

The consumption of goat’s milk products, including cheese, has increased in recent years due to their nutritional and functional properties [1,2,3]. An important element in preserving the nutritional value of dairy products is undoubtedly appropriate packaging and storage. At the time of purchase, packaging protects the contents of the product from external factors and physicochemical and microbiological changes [4]. Cheese packaging is extremely important and should take into account the type of cheese, shelf life, storage requirements, and consumer preferences. In addition, the development of new cheese packaging should take into account factors such as food safety, branding, environmental protection, and sustainability [5]. During storage, packaging protects the cheese from moisture and from spoilage caused by yeasts—*Yarrowia lipolytica*, *Kluyveromyces marxianus*, *Candida*, *Rhodotorula*, and *Cryptococcus* spp. [6]. The presence of these yeasts can cause odours, discolouration, or softening of the cheese [6]. Appropriate packaging also protects the cheese from dehydration, exposure to light, and the degradation of proteins, fats, and vitamins, and thus from deterioration [7]. Cheese stored in refrigerated conditions has a shelf life of 7 to 8 days (soft cheese) or up to 4 weeks (processed cheese) [8,9]. The shelf life of fresh white cheeses stored under aerobic conditions in active packaging can be extended by up to 3–4 weeks [10]. Vacuum-packed cheeses can extend their shelf life, as can those packed in modified atmosphere packaging (MAP). However, MAP packaging adversely affects the appearance and texture of the cheese while maintaining its sensory quality [11,12]. To prolong the freshness of cheeses, they must be stored in an appropriate form (packaging), at a low temperature, and/or in a dark place after opening. Once opened, cheeses should normally be consumed within a few hours or days, depending on the type of cheese.

How consumers store cheese at home is particularly important. Most consumers may not know the correct shelf life for cheese. In the Terpsta et al. [13] study, 22 out of 25 people did not know the correct shelf life for cheese. In general, the storage of cheese is limited to keeping it cool. The availability of many cheese-packaging materials (glass, plastic, PET, PP, PE), forms (boxes, bags), and the consumer’s lifestyle and habits—regarding refrigeration (refrigerator, storage in a low-temperature, high-humidity pantry) and organisation in the refrigerator (repackaging in smaller packages and boxes)—can change the quality and, thus, the shelf-life of cheeses. According to research by O’Callaghan and Kerry [14], a large proportion of consumers expect cheese to be kept for weeks, then days, and then months.

Goat cheeses are becoming increasingly popular with consumers and producers [15]. In the eyes of consumers, goat cheeses are natural and healthy. The consumption of such cheeses can evoke both negative and positive emotions [15]. Changing eating habits and consumer attitudes towards goat’s milk products can help to increase the demand for such products and thus increase the production of goat’s milk. When developing a cheese recipe, it is important to choose the right packaging to protect the product from spoilage. It is also important to store the product properly once it has been opened and removed from the packaging.

Therefore, the aim of this study was to evaluate changes in the water activity of goat cheeses in relation to changes in their gloss and colour during storage, depending on consumer habits. Consumers’ post-purchase behaviour with goat’s cheese was studied, including differences in cow’s cheese handling patterns, refrigerator storage time, and possible repackaging of the cheese. The selected maximum storage time of cheeses after opening the producer’s packaging and four ways of organising storage in refrigerators were the dependent variables of the experiment, which allowed the assessment of the shelf-life parameter of water activity and visual changes parameterised by the colour coordinate measurement, the calculated colour index and gloss.

The research problem was based on consumer observations regarding the repackaging of dairy products, including goat’s cheese. Consumers have reported such a problem in previous studies. An attempt was made to find out whether the repackaging of cheeses (storage in refrigerated conditions), depending on consumer preferences, has a significant effect on the change in gloss and water activity, and thus, whether the original quality of the cheeses developed by the producer is maintained. Will it be necessary in the future to indicate to the consumer the appropriate form of repackaging for a given goat’s cheese?

## 2. Materials and Methods

### 2.1. Research into the Habits of Goat’s Cheese Consumers

Participants in the original survey were consumers of different age groups (*n* = 119, aged 18–64, ME = 29) who buy and consume dairy products on a daily basis, including salad cheeses and hard cheeses made from cow’s and goat’s milk. Consumers were recruited from among students, employees, and professors at the University of Life Sciences in Poznań, as well as their families and friends. Consumers reported to consume at least three meals per day. The research tool was a validated interview questionnaire created for the purpose of the experiment. This number of people gave a general outline of consumer behaviour in society. Participation in the survey was anonymous and voluntary. The survey was carried out on paper on the premises of the University of Life Sciences in Poznań.

Consumers were asked about: (1) the type of goat’s cheese most frequently purchased (soft unripened, processed, feta salad, soft matured, hard cheese); (2) the storage time of goat’s cheese from purchase to consumption (1–7 days, 8–14 days); (3) which change they perceive as a signal inhibiting the consumption of goat’s cheese (smell, texture and consistency, colour and gloss); (4) do they treat goat’s cheese differently during storage compared to cow’s milk cheese (‘yes’; ‘no’). These were closed questions, while question 5 was an open question that complemented question 4. If the consumer answered “yes” to question 4, he could write how he repackaged the cheese.

### 2.2. Cheeses Samples

The subjects of the research were goat cheeses: (1) salad cheese and (2) hard cheese. The criterion for selecting the cheese and its type was the continuation of the research on a similar product carried out by the authors Kaczyński et al. [15]. Cheeses were purchased directly from the producer in order to minimise the time the cheeses spent in refrigerated conditions on store shelves. Control samples were products packaged and stored in refrigerated conditions and then opened on day 0 and after 7 and 14 days of storage. Soft goat’s cheese: 160 g, covering liquid 90 mL, polypropylene (PP) packaging of 250 g and with 12% fat content. Hard goat’s cheese: 150 g, wrapped in a protective atmosphere with 26% fat content. The sample of hard goat’s cheese was in the form of a block measuring 7.4 cm × 2.7 cm × 3.1 cm, where V = 61.9 cm^3^. The soft cheese samples are also in the form of a block of 10.2 cm × 2.55 cm × 2.7 cm, where V = 70.23 cm^3^.

The cheese packages were opened. The cheeses were left in the producer’s packaging or repackaged in lunchboxes (PP), aluminium foils, and plastic bags (HDPE). The lunch box was a package without access to light and was closed with a lid and latch. The volume of the lunch box was 935 cm^3^, i.e., about 7% of the surface area of the soft goat’s cheese and about 7.5% of the surface area of the hard goat’s cheese. The plastic bag (22 × 26 cm) was a transparent bag in which the cheese placed was carefully wrapped. Conversely, cheeses wrapped in aluminium foil had no access to light, and the foil adhered firmly to the sample.

Each cheese was divided into 3 equal parts and examined at different time intervals: immediately after cutting, after 7 and 14 days of storage.

Additional cheese samples were wrapped in paper made of cellulose fibre paper to test only water activity after 7 and 14 days of storage. The additional wrapping was chosen to meet the contemporary demands of consumers who prefer ecological solutions in their daily lives.

The cheeses were stored in a refrigerator (Samsung Model: RB37K63612C) at 4 ± 1 °C, humidity 40° RH. This was the correct temperature for this type of cheese [16]. The operation of the refrigerator mimicked domestic conditions in terms of the frequency of daily opening. The door was opened 40 times, which is consistent with reports by other authors [17,18]. The average time spent opening the refrigerator door was 20 min (40 × 30 s). The temperature outside the fridge was 21 °C and 46° RH. The refrigerator was not filled with other food. The experiment was repeated seven times, and each result was the average of three measurements.

### 2.3. Water Activity

The water activity was measured using an AquaLab Series 4TE instrument (Decagon Devices Inc., Pullman, Washington, DC, USA) based on pf (T), the value of the water vapor that was in equilibrium with the sample maintained at a constant level during the measurement at temperature T, and ps (T), the vapor pressure of saturated pure water at the same temperature T, as aw = pf(T)∙ps (T)^−1^. Samples of v = 15 mL provided were placed in DE 501 measurement vessels (Decagon Devices Inc., Pullman, WA, USA) and tested at 15 °C.

### 2.4. Gloss Measurement

Gloss was measured using a DT 268 gloss meter (TestAn, Gdańsk, Poland) at an angle of 60°. According to the ISO 2813 [19], the measurement geometry of 20° is used for high-gloss coatings above 70 GU (the gap angle is smaller); 60° is used for all coatings, used when testing unknown coatings; 80° is used for coatings with a lower gloss of less than 10 GU, which allows for better differentiation in the case of matte coatings. In most cases, however, measurement at an angle of 60° is most often used for both matte and glossy substrates.

### 2.5. Color Measurement

The instrumental color measurement was based on the CIELab system. A cheese sample was placed in a 2/96G/10 OG optical glass cuvette (Starna Scientific Company Ltd., Ilford, UK). The measurement was performed with a D65 light source, a 10° observation angle, with SPIN geometry using an X-Rite SP-60 camera (Grandville, MI, USA) equipped with spherical geometry (diffusive) and a measurement chamber with a DRS-811 ceramic insert. The camera was calibrated based on the black and white reference standards SP-62-162 (X-Rite, Grandville, MI, USA). The chrome (C*), whiteness index (WI), and yellowness index (YI) were calculated using the equations:

C* = [(Δa*)^2^ + (Δb*)^2^]^0.5^ (1)

WI = [(ΔL)^2^ + (Δa*)^2^ + (Δb*)^2^]^0.5^ (2)

YI = 142.86b*·L^−1^ (3)

The calculations assumed: L = 100, a* = 0, and b* = 0.

### 2.6. Water Transport

An AWC-11 water activity meter (Cobrabid, Poznań, Poland) equipped with a Rotronic probe was used to assess water transport. During 780 min of analysis, instantaneous water activity values were recorded every 10 min, and the plotted curve was divided into three areas. The first area was the constancy of the meter (water moved between the sample and the sensor in the chamber), the second area was related to the translational movement of water molecules inside the sample (beginning with an increase in the speed of water activity, and ending with reaching the equilibrium water activity; i.e., differences in water activities were less than 0.001), and the third area was related to surface processes (i.e., evacuation of water outside the sample, water activity = constant).

### 2.7. Statistical Analyses

Verification of statistical hypotheses was achieved using a level of significance of α = 0.05. The samples were evaluated by one-way analysis of variance (ANOVA) followed by Tukey’s HSD post hoc test for multiple comparisons. Data were analyzed using Statistica data analysis software (Statistica, version 13, TIBCO Software Inc., Palo Alto, CA, USA).

## 3. Results and Discussion

### 3.1. Consumer Profile

On the basis of the survey data collected, it was found that the most commonly purchased goat cheeses by consumers (Table 1) are goat salad cheese (over 36%) and hard cheese (around 25%). The least purchased cheese is melted cheese (about 8%). According to the questionnaire, the average age of consumers who buy goat’s cheese is 31 years, and it is most often bought by women (53%). The majority of consumers, 60.5%, store goat’s cheese for more than 7 days. This means that goat’s milk products can be considered “luxurious”, and consumers want to enjoy them for as long as possible. This provides a basis for analysing the shelf life of goat’s milk cheeses, depending on how they are repackaged after opening. According to questionnaire data, up to 70% of consumers treat goat’s milk cheeses differently from cow’s milk cheeses during storage. Consumers either leave the cheese in its packaging (37.3% of responses) or repackage goat’s milk cheeses in different packaging (62.7%) (Table 2). The most commonly chosen form of packaging in which consumers repack goat’s cheese is lunch boxes (30%), followed by plastic bags (HDPE) (18%) and aluminum foil (14.5%). These are the most common types of packaging in Polish households. The results show that women are more likely than men to repackage goat’s cheese (70%). Conversely, men are more likely than women to store cheese directly in the producer’s packaging (59%). Women prefer to store goat’s cheese in a plastic bag (HDPE) (100%) compared to other types of packaging. The lowest number of consumers, 14.5%, repackage cheese in aluminium foils.

The majority of consumers (around 44%) also said that colour and gloss were the most important factors when judging cheese. At the same time, they indicated that the shinier the cheese becomes during cold storage (increased gloss), the more cautious they are towards it. Secondly, consumers pay attention to structure and consistency (34.5%).

The situation is interesting when it comes to the question of open packaging from the producer. The average age is 24, and the most common choice is made by men (59%). Then, at a similar age (average 27), the product was repackaged in aluminium foils. Women are the most likely to perform this (92%). This shows how different consumer preferences are. A clear trend can be seen from the anonymous survey. The issue of repackaging the product and the way in which cheese is stored should be the subject of continuous monitoring among consumers.

### 3.2. Water Activity of Cheeses

On the basis of the tests carried out, the water activity from the time the product was opened to the end of 14 days of refrigerated storage (regardless of the packaging) ranged from 0.6379 to 0.9697 (Table 3). Typically, the water activity of most cheeses is between 0.7 and 1.0 [20]. Significant statistical differences in water activity were found for goat salad cheeses stored under refrigerated conditions, depending on the storage method. For goat’s salad cheese, the water activity on day 1 of storage was different for each storage method (*p* < 0.05). Only goat’s cheese salad had the same water activity on day 1 as in the closed packaging from the producer. During refrigerated storage, the water activity varied according to the storage method. The highest water activity after 14 days was observed for goat’s cheese in plastic bags and the lowest for goat’s cheese in aluminium foils. During refrigerated storage, the water activity increased for cheeses stored in open packaging from the producer, lunch box, and plastic bag (average of 0.0248). The greatest increase in water activity, up to 0.0378 after 14 days, was observed for cheese stored in a plastic bag (HDPE). This was probably due to the migration of water from the inside of the product to the outside and the deposition of water droplets on the plastic bag. Storing salad cheese in aluminium foils caused a decrease in water activity, which was 0.145 after 14 days. Storage in the producer’s sealed packaging does not change the water activity during refrigerated storage. Storing soft goat’s cheese in a lunch box increased the water activity by 0.016 and is slightly lower than storing cheese directly in the packaging (water activity increased by 0.0207). In addition, the higher the concentration of salt, water-soluble substances, and organic acids in the cheese, the lower the water activity. During cheese ripening, proteolytic and lipolytic processes occur, as well as water loss through evaporation. For this reason, the water activity on the outside of the product will be lower than on the inside [21,22].

However, the results for hard-ripening goat’s cheese were quite different. For almost all types of storage, the water activity was significantly reduced after 14 days of refrigerated storage. However, storage in a lunch box resulted in an increase in water activity of 0.092. The greatest change in water activity was observed when hard-ripening goat’s cheese was stored in the producer’s open packaging (reduction of 0.2360), then in aluminium foils (reduction of 0.1347), and in foil bags (reduction of 0.0631).

The type of packaging has a significant influence on the water activity of goat’s salad cheese and hard-ripening goat’s cheese. In the case of goat’s salad cheese, the best form of storage is aluminium foil. The water activity decreased significantly after 14 days of storage. The least favourable form of storage for goat’s salad cheese is a plastic bag (HDPE). For hard-ripening goat’s cheese, the best form of storage is in the producer’s open packaging, followed by aluminium foil. The worst form of storage for this type of cheese is in a lunch box, as the water activity increases significantly after 14 days of storage (*p* < 0.05).

### 3.3. Gloss of Cheeses

Storing feta salad cheese in different storage forms caused an increase in gloss at an angle of 60° during refrigerated storage in most cases (Table 4). Only the gloss of cheese stored in aluminium foils showed a significant decrease in gloss (by 2.2 GU). The increase in gloss may be due to the migration of water from the centre of the product to the outer part, thus increasing the gloss absorption of the cheese. Water activity also increases with water migration. The dependency of water evaporation from cheese depends mainly on the storage conditions and the barrier/permeability of the packaging material [23].

Different results were obtained for hard goat cheeses. When stored in refrigerated conditions, the gloss of goat’s cheese varied according to the storage method. In most cases, however, the gloss decreased significantly for cheeses stored in closed packaging from the producer, open packaging from the producer, aluminium foils, and plastic bags (HDPE). Storage of goat’s cheese in a lunch box caused a significant increase in gloss. (*p* < 0.05).

Gloss analysis showed the importance of storing cheeses in the correct form (Table 4). In the case of goat’s salad cheese, the best results in terms of the highest gloss after 14 days of refrigerated storage are obtained when it is stored in the correct way: in the producer’s open packaging (20.10 GU), in a lunch box (19.40 GU) and in the producer’s closed packaging (15.90 GU). In turn, for goat’s hard cheese, the best results are obtained when it is stored in a lunch box (11.00), in closed packaging from the producer (5.00 GU), and in a plastic bag (HDPE) (4.20 GU).

The change in gloss can be explained by the conditions prevailing in a given product. In the case of goat’s cheese, the sample first dries rapidly (the amount of whey evaporates), then there is a period of stability, and then fat droplets are formed on the product, resulting in an increase in the gloss of the sample. The situation is slightly different with goat’s hard cheese. The cheese sample is free of whey from the start; thus, the gloss decreases. During storage, the product loses moisture. In the case of “lunch box” storage, water droplets settle on the product, resulting in an increase in gloss.

In addition to colour, other properties such as shape, form, texture, and gloss are responsible for the appearance of the product [24,25]. This is also shown by recent studies where the glossiness of products can be attractive to humans. The shinier an object (material) is compared to a matte one, the more attractive it is to the human eye [26]. Such a relationship can also be observed with food products. The external appearance of a product is an important element that shapes consumer preferences [27], and gloss is one of the attributes that encourage people to buy and consume a product [28]. This is because products with a shiny surface (higher gloss) can be visually attractive to the consumer. Examples of such products are undoubtedly fruit and vegetables, as well as dairy products such as ice cream or fermented products. The more gloss a product has, the more intense the colours are, and this can encourage purchase. It has also been suggested that gloss is an indicator of the moisture content of the food surface [29]. Research by De Kerpel et al. [29] points to another aspect of gloss. The authors investigated the effect of packaging gloss on the perception of the food inside. They found that consumers can draw conclusions about the fat content of foods based on the appearance of the packaging. Glossy packaging (and a product with a certain fat content) can be a signal of fatness and may indicate negative characteristics of the product [29]. It is, therefore, important to choose the right packaging for the food. An integral function of packaging is to protect the product from spoilage and changes that may occur under the influence of external factors. It is also important to inform the consumer of the conditions under which a particular food should be stored. Studies carried out by Kampf and Nussinovitch [30] on semi-hard and dry white brine cheeses coated with different hydrocolloid films (k-carrageenan, alginate, and gellan) show that the coating contributes to better colour and gloss.

### 3.4. Colour’s Cheeses

The L*, a*, and b* parameters (Table 5) changed differently during cold storage depending on the type of storage. During this study, it was observed that the hard-ripening goat’s cheese stored in the producer’s open packaging changed its colour. The centre of the cheese became more yellow, while the edges remained unchanged. The reason for the colour change could be the oxidation of fats. On the other hand, the hard-ripening goat’s cheese stored in the lunch box was whiter after 14 days of storage (L* = 88.11) compared to the other forms of storage. Conversely, the L* parameter after 14 days of storage was lower for the hard-ripening goat’s cheese stored in the producer’s open packaging. The changes in the L*, a*, and b* parameters could be due to the lack of lipid oxidation (too short storage time). This is because, as reported by Siddique and Park [31], longer storage times (several months) at higher temperatures can increase the rate of lipid oxidation in goat’s milk cheddar cheese. Any change in the product can significantly affect its colour. In the case of salad cheeses, the proportion of white colour of the stored cheese was higher for the form of storage in closed packaging from the producer (97.21) and in open packaging from the producer (98.57).

The WI, YI, and C* parameters changed differently when goat’s salad cheese and hard-ripened cheese were stored directly in their packaging during refrigerated storage (Table 6). The WI parameter for goat’s salad cheese stored in a lunch box increased significantly by a factor of 2 (*p* < 0.05) after 14 days of storage. Conversely, the WI parameter for hard-ripened goat’s cheese in a lunch box decreased during refrigerated storage (on average by 23%). Considering that WI is of great importance for the perception of colour changes, the most advantageous way of storing goat’s cheese is in the producer’s open packaging. This parameter is significantly lower (*p* < 0.05) after 14 days of storage. On the other hand, when this cheese is stored in a lunch box, the WI parameter is significantly higher after 14 days of storage (*p* < 0.05). For goat’s hard-ripening cheese, it is best to store the cheese in a lunch box. The WI parameter is significantly lower after 14 days of storage (*p* < 0.05). The worst way to store hard-ripening goat’s cheese is in the producer’s open packaging. The change in WI is about two times higher after 14 days of storage (*p* < 0.05).

As shown by the studies of Alabdulkarim et al. [32] on traditional Oggt cheeses, appropriate packaging affects the growth and development of microorganisms, including LAB bacteria, during cold storage. They showed that oxygen permeability and permeability characteristics were higher for LDPE packaging, and water vapour permeability was higher for HDPE packaging. However, their studies showed that storing OGT cheese in different types of packaging did not significantly affect the development of yeasts and moulds. In our studies, based on observations, we found that mould developed faster in hard-ripening goat cheeses stored in glass packaging and in soft cheeses stored in foil bags than in other packaging.

^a^Parameters: L* lightness, a* − green/+ red color, b* − blue/+ yellow

### 3.5. Molecular Dynamics of Water and Water Transport

**Figure 1:**
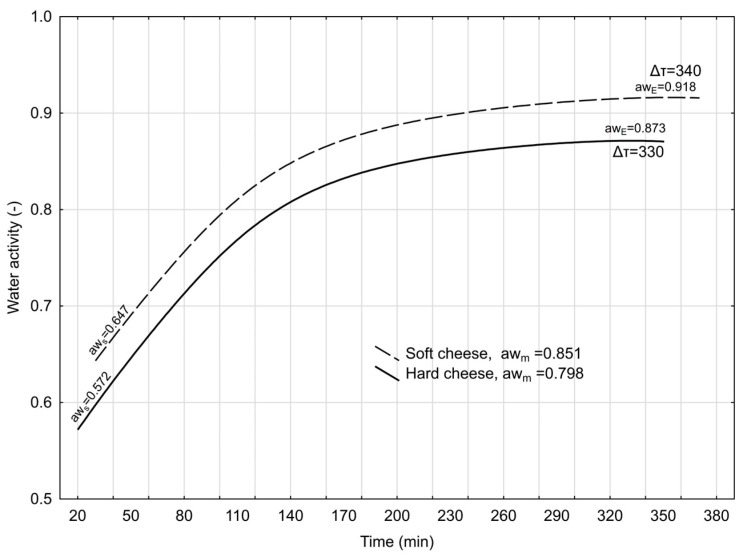
Translational movement of water in soft goat cheese and hard goat cheese. aw_m_ = mean water activity translation area; Δτ = duration of translational water movement in minutes; aw_s_ = initial water activity; aw_E_ = final water activity.

Figure 1 shows the analysis of the change in water activity over time (translational water movement) for soft and hard goat cheeses.

The kinetics of water molecule movement were faster for hard cheese (0.97 × 10^−3^ min, Table 7) than for soft cheese. The initial water activity for soft cheese was 0.647 (in 30 min), whereas for hard cheese, it was 0.572 (in 20 min). The end of the transformation period was higher for soft cheese (370 min, Table 7) than for hard cheese (350 min, Table 7), corresponding to 0.918 and 0.873 water activities, respectively.

The degree of water dispersion in fat strongly influences water activity. The smaller the water droplet, the higher the vapour pressure above its surface and, thus, the higher the water activity [33]. As shown in Figure 1, the water activity after 370 min was lower for hard goat’s cheese (0.873). As stated by Tomaszewska-Gras et al. [22], the lower the moisture content of the cheese, the lower the water activity value. In addition, salting and ripening undoubtedly have an effect on the reduction in water activity. These two factors are responsible for a greater decrease in activity and are closely related [22]. This shows how all factors, from cheese production to the way cheese is repackaged and stored by consumers, are important in keeping cheese fresh for as long as possible.

### 3.6. The Relationship Between Cold Storage of Cheese and Parameters

In order to verify this hypothesis, an analysis was made of the correlation between water activity and the type of storage of goat’s cheese according to consumer preferences. As can be seen (Table 8), the storage method only influences the change in gloss in the case of goat’s salad cheese and the a* parameter for hard cheeses. On the other hand, the influence of the water activity of hard goat’s cheese affected the parameters L*, a*, and b*. The storage method has the least effect on the parameter b* for hard cheese.

Taking into account the data analysed, the strongest dependencies occur between the water activity of hard cheeses and the gloss and colour parameters. Therefore, for goat’s hard cheese, it should be of the utmost importance for cheese producers to maintain the original quality of the cheese when repackaging the opened cheese. This statistical analysis also shows that in this type of research, it is worth carrying out a correlation analysis, as this gives more accurate information than if each factor was considered separately.

### 3.7. Strategic Foresight: Ecological Consumer

In a changing environment, every consumer’s primary goal should be to eliminate the storage of food (including as many dairy products as possible) in plastic (PP) packaging. However, consumers choose practical solutions based on personal experience (visual and tactile). Most consumers (30.1%) use a lunch box to store food. Such a lunch box does not cause any negative changes in the food. It fulfils its role and protects the product from the external environment. However, such packaging may be destroyed in the future and thrown into the rubbish, which has a negative impact on the environment. In turn, as reported in research by Langley et al. [34], it is more important for the consumer to reduce the amount of packaging, including plastic, than to reduce food waste.

Storing cheese in paper packaging can be beneficial to the environment by removing plastic from the environment. Unfortunately, storing cheese, especially soft cheese, in paper can be problematic due to its poor barrier properties [35]. Most consumers have ordinary paper in their homes, which is made from cellulose fibres coated with various fats or silicone. Leakage of whey can cause such paper packaging to become wet and stick to the product. Although the water activity (Table 9) of soft goat’s cheese decreased by 2 and 5% after 7 and 14 days of storage (due to the absorption of excess whey from the product), respectively, this did not visually encourage re-consumption of the product. In the case of hard goat’s cheese, the water activity in cellulose paper increased by 2% and 4% after 7 and 14 days of storage, respectively.

It would, therefore, be necessary to work together to develop ideal solutions that would eliminate plastic and provide an ideal barrier for dairy products. Such solutions could be implemented at every stage of a given product, from the producer (when he packages a given food product) to the consumer—including his forms of storing dairy products.

### 3.8. Theoretical and Practical Implications of This Study

In order to better understand the research problem, present the results and conduct this discussion, it is necessary to realise the importance of maintaining the original freshness of cheeses. Changing consumer habits and demands are forcing producers to develop new types of packaging to ensure the freshness of a given cheese. Cheese is highly susceptible to physical, chemical, and biochemical spoilage. The growth of mesophilic and psychotropic bacteria, lipid oxidation, and enzymatic degradation determine the quality and stability of cheese [9]. Several factors can prolong the shelf life of cheese, such as the MAP system, temperature control during storage, and humidity. However, it all depends on the consumer and how they store food, including cheese. It is important to educate the consumer on how to repackage cheese after opening, as this determines the extension of the freshness of the cheese. Therefore, in practice, the consumer should have a clear signal from the producer as to which repackaging (storage) practice is best. Efforts should also be made to ensure that such packaging (including the packaging in which the product is delivered by the producer after opening) does not harm the natural environment.

## 4. Conclusions

Based on the study’s preferences for storing open goat’s cheese in refrigerated conditions, it was shown that the best packaging for storing open goat’s cheese, such as feta cheese, was aluminium foils, and the least popular was foil bags. For hard goat cheeses, the best packaging was found to be the producer’s packaging. It was also shown that there is a correlation between the water activity and the parameters depending on the storage method (mainly for gloss and L*; a* and b*). The survey shows that about 44% of consumers have noticed a change in the colour and gloss of the cheese, which is one of the signals that discourage the consumption of goat’s cheese. Most consumers stated that the change in colour and gloss was one of the most important characteristics of cheese quality and could, therefore, indicate its possible spoilage. Any change in the colour of the product can be a signal to the consumer, indicating the freshness of the product. However, the observation of gloss can complement the colour change. The research carried out is important for cheese producers and packaging producers. It can help to increase consumption and sales of cheeses by improving their image. Choosing the type of packaging for goat’s cheese according to the consumer’s preferences (habits) can help maintain the original quality of the cheese developed by the producer. The research results can help to develop guidelines for producers, not only in the area of packaging design but also in labelling (by including appropriate information for the consumer on how to store cheeses in the refrigerator and the range of appropriate temperatures).

Given the changing habits of consumers (always on the go), it will be difficult to predict what happens to cheeses when they are opened and how they are stored. The key task for future research is, therefore, to carry out regular consumer surveys. The results of this study show that young people ($\;\bar{x}\;$ = 24) leave the cheese in the producer’s packaging after opening. This behaviour may be repeated in the future and passed on from generation to generation. Therefore, it is necessary to choose (develop) a packaging method that would preserve the original quality of the cheeses when stored in this way in refrigerated conditions. Such a solution should also be beneficial to the natural environment. It is also worth extending the research in the future to include the microscopic structure of cheeses stored in different conditions, as well as analysing the cheeses from a microbiological point of view.

## Figures and Tables

**Table 1:** Responses to the questions asked from the questionnaire.

Dependence	The Most Frequently Purchased Type of Goat Cheese					Storage Time of Goat Cheese		A Signal Inhibiting The Consumption of Goat Cheese			Do You Handle Goat Cheese Differently Than Goat Milk Cheese During Storage?	
	Soft Unripened	Melted Cheese	Goat’s Salad Cheese	Soft Ripening	Hard Cheese	1–7 Days	8–14 Days	Odour	Structure and Consistency	Color and Gloss	Yes	No
Number of responses	12.6%	8.4%	36.1%	17.7%	25.2%	39.5%	60.5%	21.8%	34.5%	43.7%	69.7%	30.3%
$\bar{x}$ age (age from–to)	29 (18–41)	43 (38–47)	31 (19–50)	32 (19–57)	32 (18–64)	44 (28–64)	24 (18–36)	35 (29–45)	43 (20–64)	22 (18–28)	36 (18–64)	29 (18–39)
Sex	F = 67% M = 33%	F = 70% M = 30%	F = 53% M = 47%	F = 67% M = 33%	F = 70% M = 30%	F = 66% M = 34%	M = 56% F = 44%	F = 62% M = 38%	F = 66% M = 34%	F = 62% M = 38%	F = 70% M = 30%	M = 53% F = 47%
Median age	28	44.5	32	25	22	41	24	35	44	21.5	27	29

$\;\bar{x}\;$ —mean; F—Female; M—Male.

**Table 2:** Methods of refrigerated cheese storage reported by respondents who handle cheese differently from cow’s milk cheese.

Dependence	In Open Packaging from the Producer	Lunch Box (PP)	Aluminium Foil	Plastic Bag (HDPE)
Number of responses	37.3%	30.1%	14.5%	18.1%
$\bar{x}$ age	24 (18–50)	37 (24–55)	27 (18–58)	54 (25–64)
Sex	M = 59% F = 41%	F = 76% M = 24%	F = 92% M = 8%	F = 100% M = 0%
Median age	24	36	19.5	57

$\;\bar{x}\;$—mean; F—Female; M—Male.

**Table 3:** Water activity of goat’s cheese stored using different storage methods under refrigerated conditions.

Storage Method	Day	Salad Cheese	Hard Cheese
In Closed the Packaging From the Producer *	1	0.9309 ± 0.0016 ^aA^	0.8744 ± 0.0033 ^aB^
	7	0.9320 ± 0.0005 ^aB^	0.8765 ± 0.0011 ^aB^
	14	0.9330 ± 0.0017 ^aB^	0.8781 ± 0.0034 ^aD^
In Open Packaging From the Producer	1	0.9397 ± 0.0007 ^aB^	0.8739 ± 0.0017 ^bB^
	7	0.9564 ± 0.0009 ^bC^	0.8686 ± 0.0039 ^bA^
	14	0.9604 ± 0.0011 ^cC^	0.6379 ± 0.0025 ^aA^
Lunch Box (PP)	1	0.9451 ± 0.0012 ^aC^	0.8306 ± 0.0011 ^aA^
	7	0.9559 ± 0.0005 ^bC^	0.8745 ± 0.0013 ^bB^
	14	0.9611 ± 0.0015 ^cC^	0.9226 ± 0.0013 ^cE^
Aluminium Foil	1	0.9544 ± 0.0006 ^cD^	0.9437 ± 0.0022 ^cD^
	7	0.9099 ± 0.0021 ^bA^	0.9279 ± 0.0012 ^bD^
	14	0.8094 ± 0.0011 ^aA^	0.8090 ± 0.0010 ^aB^
Plastic Bag (HDPE)	1	0.9319 ± 0.0014 ^aA^	0.9229 ± 0.0010 ^cC^
	7	0.9544 ± 0.0017 ^bC^	0.9098 ± 0.0015 ^bC^
	14	0.9697 ± 0.0021 ^cD^	0.8598 ± 0.0054 ^aC^

* Control samples. Samples were independent of storage time. Small different letters in a column for the same storage method indicate the effect of cheese storage time, and different capital letters in a column for the same storage time indicate the effect of cheese storage method at the level α = 0.05.

**Table 4:** Gloss (GU) of goat’s cheese stored under different conditions.

Storage Method	Day	Salad Cheese	Hard Cheese
		60°	
In Closed the Packaging From the Producer *	1	14.65 ± 0.10 ^aD^	5.65 ± 0.05 ^bC^
	7	15.05 ± 0.09 ^bD^	5.50 ± 0.05 ^bD^
	14	15.90 ± 0.05 ^cC^	5.00 ± 0.30 ^aD^
In open Packaging From the Producer	1	14.80 ± 0.09 ^aD^	5.70 ± 0.05 ^cC^
	7	17.50 ± 0.13 ^bE^	3.10 ± 0.05 ^bA^
	14	20.10 ± 0.10 ^cE^	2.40 ± 0.05 ^aA^
Lunch Box (PP)	1	10.30 ± 0.33 ^aC^	2.80 ± 0.09 ^aA^
	7	12.10 ± 0.13 ^bC^	4.10 ± 0.18 ^bB^
	14	19.40 ± 0.18 ^cD^	11.00 ± 0.22 ^cE^
Aluminium Foil	1	2.80 ± 0.05 ^bB^	4.50 ± 0.10 ^bB^
	7	0.70 ± 0.00 ^aA^	4.40 ± 0.15 ^bB^
	14	0.60 ± 0.05 ^aA^	3.40 ± 0.18 ^aB^
Plastic Bag (HDPE)	1	2.10 ± 0.13 ^aA^	5.70 ± 0.22 ^cC^
	7	6.70 ± 0.15 ^bB^	4.80 ± 0.09 ^bC^
	14	7.20 ± 0.13 ^cB^	4.20 ± 0.09 ^aC^

* Control samples. Samples were independent of storage time. Small different letters in a column for the same storage method indicate the effect of cheese storage time, and different capital letters in a column for the same storage time indicate the effect of cheese storage method at the level α = 0.05.

**Table 5:** Assessment of the color of the salad cheeses.

Storage Method	Day	Salad Cheese			Hard Cheese		
		L*	a*	b*	L*	a*	b*
In Closed the Packaging From the Producer *	1	96.40 ± 0.23 ^aA^	−1.05 ± 0.15 ^aA^	5.40 ± 0.03 ^aB^	86.25 ± 0.10 ^aC^	−2.35 ± 0.26 ^aA^	10.96 ± 0.09 ^aB^
	7	96.80 ± 0.63 ^aB^	−1.06 ± 0.06 ^aB^	6.00 ± 0.05 ^bA^	85.88 ± 0.60 ^aC^	−2.95 ± 0.48 ^aB^	11.20 ± 0.15 ^abB^
	14	97.21 ± 0.43 ^aCD^	−1.07 ± 0.11 ^aAB^	6.10 ± 0.06 ^bA^	85.55 ± 1.10 ^aC^	−3.00 ± 0.15 ^aB^	11.25 ± 0.09 ^bB^
In open Packaging From the Producer	1	96.51 ± 0.17 ^aA^	−1.07 ± 0.02 ^aA^	5.50 ± 0.13 ^aBC^	85.98 ± 0.27 ^cC^	−2.30 ± 0.19 ^aA^	10.92 ± 0.12 ^aB^
	7	97.01 ± 0.17 ^aB^	−1.17 ± 0.08 ^aAB^	6.49 ± 0.14 ^cBC^	65.04 ± 1.01 ^aA^	−3.68 ± 0.09 ^bA^	11.44 ± 0.28 ^bB^
	14	98.57 ± 0.94 ^bD^	−1.06 ± 0.06 ^aAB^	6.14 ± 0.13 ^bA^	66.65 ± 0.37 ^bA^	−3.88 ± 0.27 ^bA^	11.43 ± 0.07 ^bB^
Lunch Box (PP)	1	98.68 ± 0.59 ^cB^	−1.01 ± 0.29 ^aA^	3.11 ± 0.66 ^aA^	80.48 ± 1.63 ^aA^	−2.20 ± 0.13 ^bA^	10.55 ± 0.12 ^aA^
	7	96.61 ± 0.11 ^bB^	−1.04 ± 0.11 ^aB^	6.91 ± 0.10 ^bD^	86.10 ± 0.64 ^bC^	−2.27 ± 0.11 ^abC^	11.04 ± 0.11 ^bAB^
	14	94.07 ± 0.30 ^aA^	−1.27 ± 0.09 ^aA^	7.61 ± 0.08 ^bB^	88.11 ± 0.20 ^bD^	−2.49 ± 0.08 ^aC^	11.20 ± 0.15 ^bB^
Aluminium Foil	1	96.65 ± 0.55 ^bA^	−1.10 ± 0.13 ^abA^	5.99 ± 0.06 ^aBC^	85.38 ± 0.11 ^aBC^	−2.17 ± 0.05 ^aA^	10.53 ± 0.09 ^aA^
	7	95.47 ± 0.29 ^aA^	−1.29 ± 0.07 ^bA^	6.72 ± 0.08 ^abCD^	85.71 ± 0.67 ^aC^	−2.15 ± 0.02 ^aC^	10.64 ± 0.06 ^aA^
	14	96.33 ± 0.32 ^abBC^	−0.98 ± 0.04 ^aB^	6.16 ± 0.41 ^bA^	85.75 ± 0.44 ^aC^	−2.17 ± 0.02 ^aC^	11.37 ± 0.11 ^bB^
Plastic Bag (HDPE)	1	96.32 ± 0.20 ^aA^	−1.13 ± 0.03 ^aA^	6.28 ± 0.04 ^aC^	83.83 ± 0.33 ^aB^	−2.35 ± 0.10 ^bA^	10.89 ± 0.04 ^bB^
	7	96.31 ± 0.20 ^aAB^	−1.09 ± 0.01 ^aB^	6.27 ± 0.03 ^aB^	83.90 ± 0.06 ^aB^	−2.17 ± 0.02 ^aC^	10.65 ± 0.05 ^aA^
	14	96.40 ± 0.23 ^aA^	−1.05 ± 0.15 ^aA^	5.40 ± 0.03 ^aB^	86.25 ± 0.10 ^aC^	−2.35 ± 0.26 ^aA^	10.96 ± 0.09 ^aB^

* Control samples. Samples were independent of storage time. Small different letters in a column for the same storage method indicate the effect of cheese storage time, and different capital letters in a column for the same storage time indicate the effect of cheese storage method at the level α = 0.05.

**Table 6:** Assessment of the color of the salad cheeses with parameters.

Storage Method	Day	Salad Cheese			Hard Cheese		
		C*	WI	YI	C*	WI	YI
In Closed the Packaging From the Producer *	1	5.50 ± 0.05 ^aB^	6.57 ± 0.10 ^aB^	8.00 ± 0.03 ^aB^	11.21 ± 0.14 ^aB^	17.74 ± 0.14 ^aA^	18.15 ± 0.16 ^aAB^
	7	6.09 ± 0.04 ^bA^	6.88 ± 0.32 ^aA^	8.85 ± 0.12 b^A^	11.59 ± 0.10 ^bB^	18.27 ± 0.52 ^aAB^	18.63 ± 0.37 ^aB^
	14	6.19 ± 0.04 ^bA^	6.79 ± 0.21 ^aAB^	8.96 ± 0.12 b^A^	11.64 ± 0.11 ^bB^	18.57 ± 0.88 ^aB^	18.79 ± 0.27 ^aB^
In open Packaging From the Producer	1	5.60 ± 0.13 ^aBC^	6.60 ± 0.06 ^aB^	8.14 ± 0.18 ^aB^	11.16 ± 0.15 ^aB^	17.92 ± 0.12 ^aAB^	18.14 ± 0.15 ^aAB^
	7	6.59 ± 0.15 ^cB^	7.24 ± 0.13 ^bA^	9.56 ± 0.20 ^cB^	12.02 ± 0.25 ^bC^	36.97 ± 0.91 ^cC^	25.13 ± 0.44 ^bC^
	14	6.23 ± 0.14 ^bA^	6.39 ± 0.34 ^aA^	8.90 ± 0.27 ^bA^	12.07 ± 0.07 ^bC^	35.47 ± 0.34 ^bC^	24.50 ± 0.03 ^bC^
Lunch Box (PP)	1	3.27 ± 0.70 ^aA^	3.53 ± 0.44 ^aA^	4.50 ± 0.94 ^aA^	10.78 ± 0.14 ^aA^	22.31 ± 1.40 ^bC^	18.73 ± 0.36 ^aC^
	7	6.99 ± 0.11 ^bC^	7.77 ± 0.14 ^bBC^	10.22 ± 0.15 ^bC^	11.27 ± 0.10 ^bB^	17.90 ± 0.44 ^aA^	18.32 ± 0.06 ^aAB^
	14	7.72 ± 0.08 ^bB^	9.73 ± 0.19 ^cD^	11.56 ± 0.13 ^bB^	11.47 ± 0.16 ^bB^	16.52 ± 0.06 ^aA^	18.16 ± 0.21 ^aA^
Aluminium Foil	1	6.09 ± 0.08 ^aC^	6.95 ± 0.25 ^aBC^	8.85 ± 0.08 ^aBC^	10.75 ± 0.09 ^aA^	18.15 ± 0.09 ^aAB^	17.62 ± 0.14 ^aA^
	7	6.84 ± 0.06 ^bC^	8.21 ± 0.13 ^bC^	10.06 ± 0.09 ^bC^	10.86 ± 0.07 ^aA^	17.95 ± 0.50 ^aAB^	17.73 ± 0.05 ^aA^
	14	6.24 ± 0.40 ^aA^	7.24 ± 0.29 ^aBC^	9.14 ± 0.59 ^aA^	11.58 ± 0.10 ^bB^	18.36 ± 0.28 ^aB^	18.94 ± 0.08 ^bB^
Plastic Bag (HDPE)	1	6.38 ± 0.04 ^aC^	7.37 ± 0.07 ^aC^	9.31 ± 0.04 ^aC^	11.14 ± 0.05 ^bB^	19.64 ± 0.26 ^aB^	18.56 ± 0.08 ^bBC^
	7	6.36 ± 0.02 ^aB^	7.36 ± 0.11 ^aAB^	9.30 ± 0.05 ^aB^	10.87 ± 0.05 ^aA^	19.43 ± 0.06 ^aB^	18.13 ± 0.09 ^aAB^
	14	6.54 ± 0.02 ^bA^	7.79 ± 0.17 ^bC^	9.61 ± 0.03 ^bA^	11.04 ± 0.01 ^bA^	19.54 ± 0.18 ^aB^	18.29 ± 0.06 ^aA^

* Control samples. Samples were independent of storage time. Small different letters in a column for the same storage method indicate the effect of cheese storage time, and different capital letters in a column for the same storage time indicate the effect of cheese storage method at the level α = 0.05.

**Table 7:** Kinetics of translational motion of water molecules in goat cheese.

Parameter ^1^	Soft Cheese	Hard Cheese
τe (min)	370	350
Δaw	0.271	0.301
Vm (min × 10^−3^)	0.60	0.97
τm (min)	40	30

^1^ τe = the end of the translation period; Δaw = water activity from the start of the translation phase to the start of surface conduction; Vm = maximum rate of change in water activity over time; τm = time to reach maximum speed.

**Table 8:** Correlation between water activity and storage method of goat cheeses according to consumer preferences.

Parameter	Water Activities of Salad Cheese	Water Activities of Hard Cheese	Storage Method
Gloss 60° Salad Cheese	0.276145	−0.388597	−0.765484
L* salad cheese	0.097865	−0.655370	−0.285217
a* salad cheese	−0.234431	−0.451044	−0.139693
b* salad cheese	0.135504	0.225268	0.080875
Gloss 60° Hard Cheese	−0.235318	0.516178	−0.043522
L* hard cheese	−0.693000	0.616603	0.219597
a* hard cheese	−0.550992	0.605076	0.543180
b* hard cheese	0.389241	−0.521038	−0.481222

Underlined values indicate the presence of correlation. The correlation coefficient is marked at *p* < 0.05.

**Table 9:** Water activity of goat’s cheese wrapped in cellulose fibre paper (*n* = 3).

	Water Activity in 1 Day	Water Activity in 7 Days	Water Activity in 14 Days
salad cheese	0.9311 ± 0.0014 ^c^	0.9125 ± 0.0011 ^b^	0.8845 ± 0.0013 ^a^
hard cheese	0.8749 ± 0.0025 ^a^	0.8924 ± 0.0008 ^b^	0.9099 ± 0.0047 ^c^

Means within a row with different superscripts differ (*p* < 0.05).

## Data Availability

The original contributions presented in the study are included in the article, further inquiries can be directed to the corresponding author.
